# Supplementary material for: TATA and paused promoters active in differentiated tissues have distinct expression characteristics
Source: Mol Syst Biol. 2021 Feb 5;17(2):e9866. doi: 10.15252/msb.20209866 (PMC7863008; doi:10.15252/msb.20209866)
Supplement: Supplementary file 1 — Appendix [file MSB-17-e9866-s001.pdf]

Table of Contents

Figure S1: scRNA-seq experiments provide reproducible and high-quality gene expression profiles 2

Figure S2: Expression profiles of marker genes of the clusters identified in the scRNA-seq data 3

Figure S3: Tissue-specific Pol II ChIP-seq shows specificity and correlates with the scRNA-seq expression profile 4

Figure S4: TATA gene expression increases over time 4

Figure S5: TATA genes often occur in clusters and are short 5

Table S1: Tissue-specific gal4 lines used in the INTACT experiments 6

Table S2: Promoter motifs. Motifs are scored with zero mismatches in the specified windows relative to the TSS ... 6

Figure S1

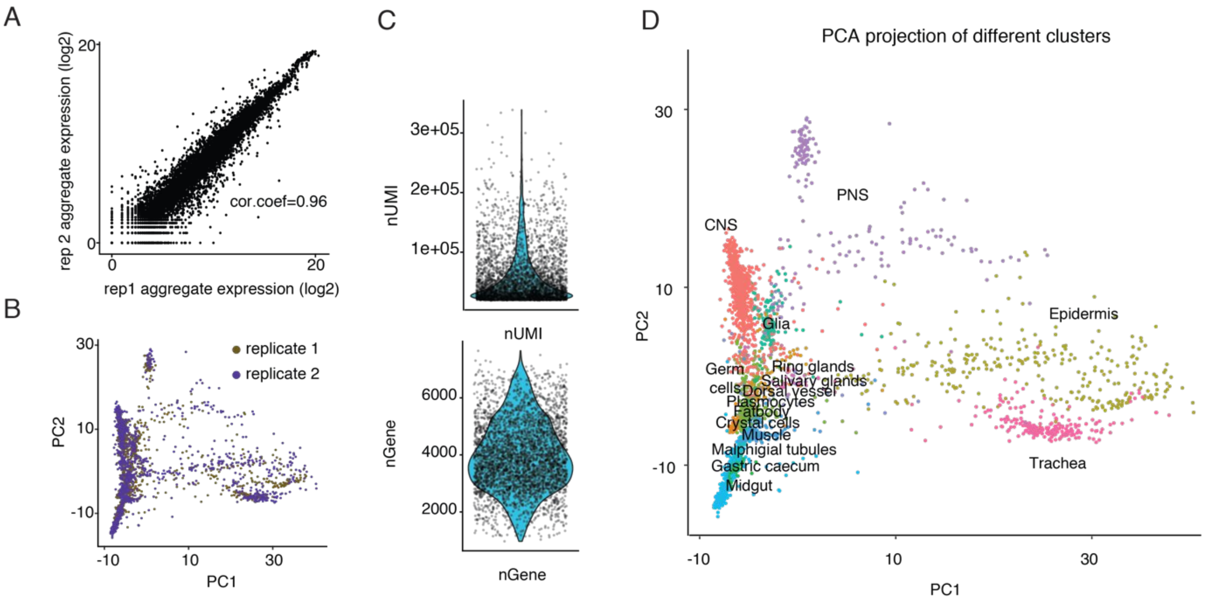

Figure S1: scRNA-seq experiments provide reproducible and high-quality gene expression profiles. (A) A high Pearson correlation coefficient of gene expression levels was observed between single-cell replicates, computed as the sum of read counts for each gene across all cells in both replicates. (B) PCA projection of the scRNA-seq data from both replicates shows consistency between the two replicates. (C) Number of Unique Molecular identifiers (UMI) and number of genes captured per cell are shown. On average, about 4,000 genes per cell were captured. (D) PCA projection of the scRNA-seq data shows that tissues of similar origin cluster together, which indicates that the main sources of variations in the scRNA-seq data are biologically significant.

Figure S2

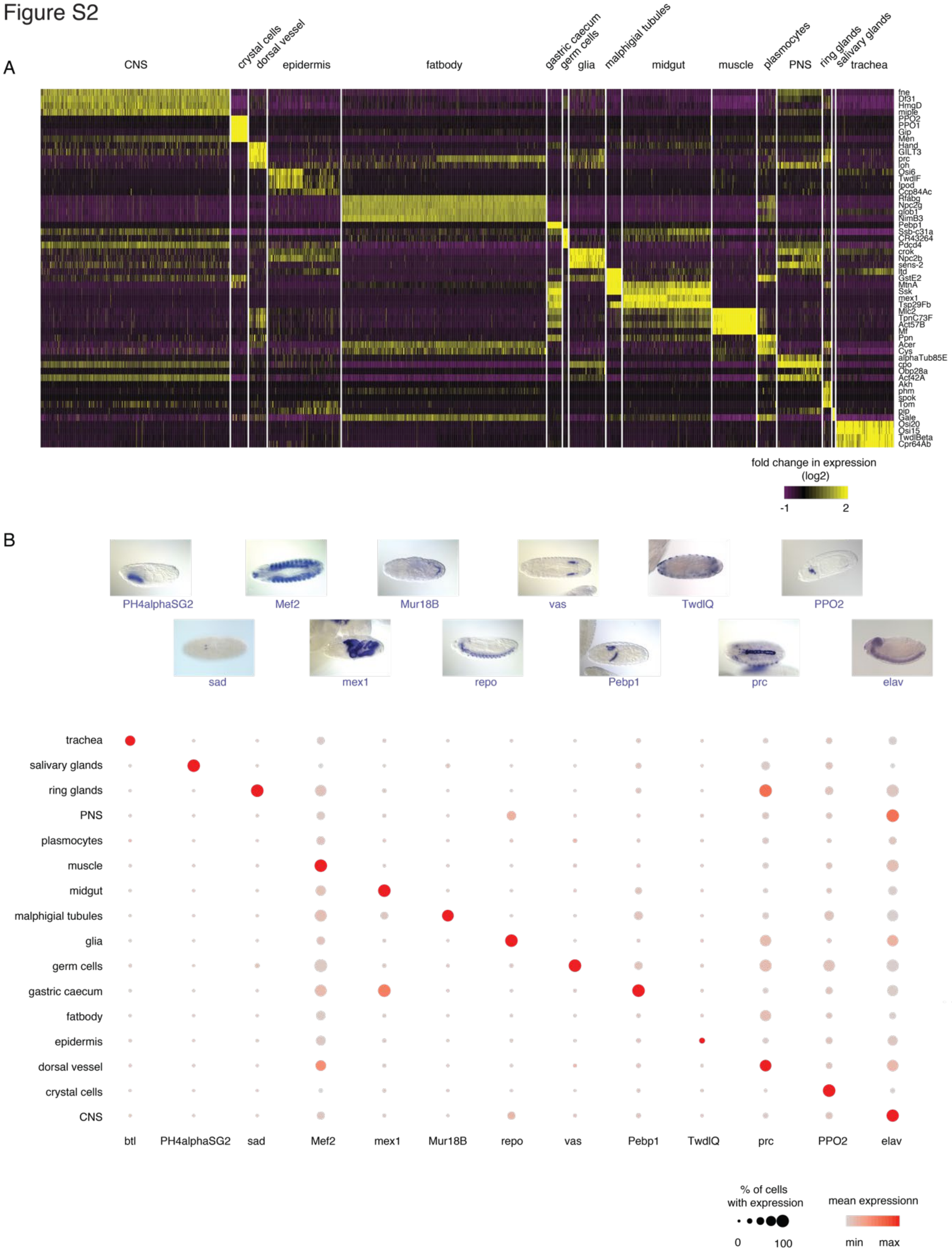

Figure S2: Expression profiles of marker genes of the clusters identified in the scRNA-seq data. (A) Heat map of *de-novo* identified marker genes. Shown are the top four differentially expressed genes in each tissue (Wilcoxon rank sum test with P value < 0.01), expression in at least 5% of cells in at least one group). (B) Among them are many known marker genes based on previous studies and *in situ* hybridization from BDGP, as shown in the dot plot. The size of the dot represents the frequency of cells in a tissue with expression and the color intensity represents the mean expression in the tissue.

Figure S3

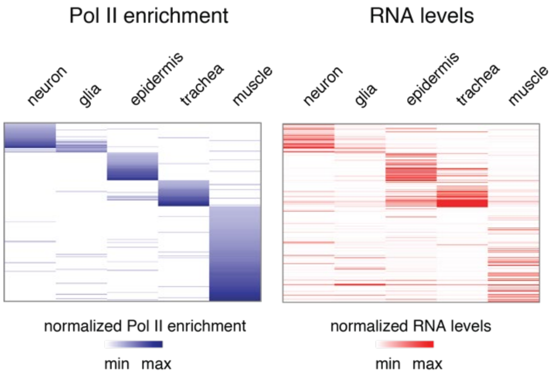

Figure S3: Tissue-specific Pol II ChIP-seq shows specificity and correlates with the scRNA-seq expression profile.
The specificity of the tissue-specific Pol II ChIP-seq was evaluated by analyzing the correlation between the Pol II occupancy pattern for a gene and its corresponding expression profile in the scRNA-seq data. The analysis shown here was restricted to genes with Pol II occupancy in a single tissue (calculated around a window starting from the TSS and ending 200 bp downstream). There is a clear visual correspondence between the Pol II occupancy and the scRNA-seq gene expression profile. For 77% of the genes, the tissue with the highest Pol II occupancy was also the tissue with the highest RNA levels.

Figure S4

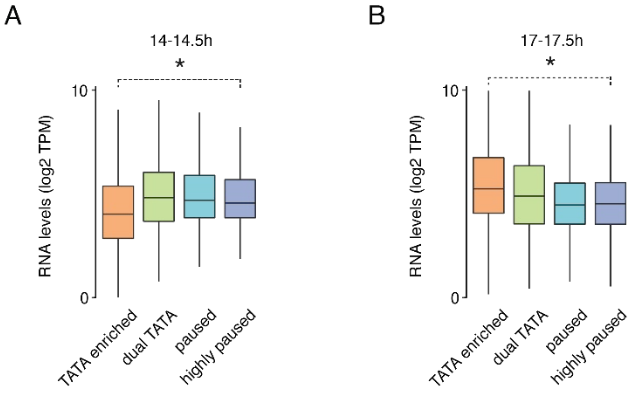

Figure S4: TATA gene expression increases over time. (A & B) RNA levels (log2 TPM) from (A) 14-14.5 h and (B) 17-17.5 h embryos at the different effector gene groups are shown. (A) The TATA genes are expressed at levels lower than the paused genes at 14-14.5 h (Wilcoxon two-sided test, *P $<10^{-11}$). (B) By 17-17.5 h, the TATA genes are expressed at levels higher than the paused genes (Wilcoxon two-sided test, *P $<10^{-9}$).

Figure S5

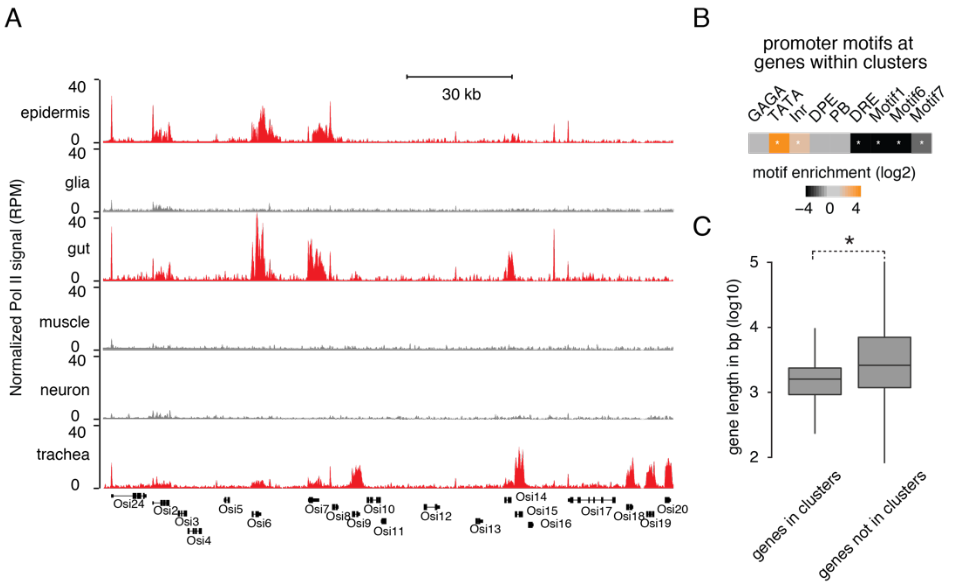

Figure S5: TATA genes often occur in clusters and are short. (A) Read-normalized Pol II signals (RPM) at a gene cluster are shown. (B) Promoter elements enriched at genes present in clusters were compared to all other genes. Genes present in clusters are enriched for the TATA motif (*P < 0.05). (C) Total gene lengths of the genes that are present in clusters were compared to all other genes. Genes present in clusters are shorter than the genes which are not present in clusters (Wilcoxon two-sided test, *P <10^{-15}).

tissue	tissue-specific Gal4 driver line
Neuron	w[*]; P{w[+mC]=GAL4-elav.L}3, p[UAS-3xFLAG-blrp-mCherry- RanGap, UAS-BirA)6
Glia	w[*]; P{w[+m*]=GAL4}repo, p[UAS-3xFLAG-blrp-mCherry- RanGap, UAS-BirA)6/TM6Tb
Trachea	w[*]; P{w[+mC]=GAL4-btl.S}2, P{w[+m*]=lacZ-un8}276, p[UAS-3xFLAG-blrp-mCherry- RanGap, UAS-BirA)5/Cyo
Epidermis	P{w[+mW.hs]=GawB}112A, w[*],p[UAS-3xFLAG-blrp-mCherry- RanGap, UAS-BirA)6
Muscle	w[*]; P{w[+mC]=GAL4-Mef2.R}3, p[UAS-3xFLAG-blrp-mCherry- RanGap, UAS-BirA)6/TM3Sb
Gut (Foregut and Hindgut)	w[*]; P{GawB}NP3084 , p[UAS-3xFLAG-blrp-mCherry- RanGap, UAS-BirA)5/Cyo

Table S1: Tissue-specific gal4 lines used in the INTACT experiments.

name	motif	window_start (bp)	window_end (bp)
DPE	KCGGTTSK	20	40
DRE	WATCGATW	-250	0
GAGA	GAGAG	-200	0
Inr	TCAKTY	-10	10
MTE	CSARCSSA	10	30
PB	KCGRWCG	20	40
TATA	STATAWAWR	-40	-20
Motif1	YGGTCACACTR	-250	0
Motif6	YRGTATWTTY	-250	0
Motif7	CAKCNCTR	-250	0
BREd	RTDKKKK	-30	-10
BREu	SSRCGCC	-50	-30

Table S2: Promoter motifs. Motifs are scored with zero mismatches in the specified windows relative to the TSS.
