# Supplementary material for: TATA and paused promoters active in differentiated tissues have distinct expression characteristics
Source: Mol Syst Biol. 2021 Feb 5;17(2):e9866. doi: 10.15252/msb.20209866 (PMC7863008; doi:10.15252/msb.20209866)
Supplement: Supplementary file 2 — Expanded View Figures PDF [file MSB-17-e9866-s002.pdf]

Expanded View Figures

Figure EV1. Functional categories and categorization of late-induced effector genes.

A, B GO term enrichments were calculated for Biological Processes. For each gene group and each GO term, the enrichment was calculated as the fraction of genes in a group associated with a GO term over the fraction of all genes with the same GO term. The statistical significance was calculated using the hypergeometric test. GO terms associated with less than five genes were not included in the analysis. Top representative terms are shown in the bar plot. (A) Identified effector genes are enriched for expected GO terms. (B) GO term enrichments for the TATA group and the highly paused group effector genes were calculated separately. While there are some differences between the groups, both groups have functional categories expected for effector genes.

C The identified effector genes were enriched for promoter elements found at tissue-specific genes (focused promoter elements) and are depleted for motifs associated with housekeeping genes (broad promoter elements). A star denotes significance with a Fisher's exact test, (*$P < 0.05$).

D Pausing indices ($\log_2$), the ratio of Pol II at the promoter vs the gene body, are shown for the different effector gene groups. The pausing indices of genes from the TATA group are significantly lower than that of the highly paused group (Wilcoxon two-sided test, *$P < 10^{-15}$).

E RNA levels ($\log_2$ TPM) from 14 to 17 h whole embryos at the different effector gene groups are shown. The TATA genes are expressed at levels comparable to the paused genes.

F The sequence heat map plot shows motif differences between the different gene groups. Information content at each position is plotted as a sequence logo.

G Pausing index and total gene lengths of genes, of TATA and highly paused effector genes, developmentally paused and housekeeping groups are shown. The pausing indices of genes from the TATA group are significantly lower than that of the highly paused group (Wilcoxon two-sided test, *$P < 10^{-15}$; left panel). TATA genes are generally shorter than highly paused genes (Wilcoxon two-sided test, *$P < 10^{-15}$; right panel).

H Promoter shape values are based on the CAGE data (Sigalova *et al*, 2020). Promoters of TATA-enriched, highly paused and developmentally paused genes groups are primarily narrow, while the housekeeping gene promoters are primarily broad. Box plots in all panels show the median as the central line, the first and the third quartiles as the box, and the upper and lower whiskers extend from the quartile box to the largest/smallest value within 1.5 times of the interquartile range.

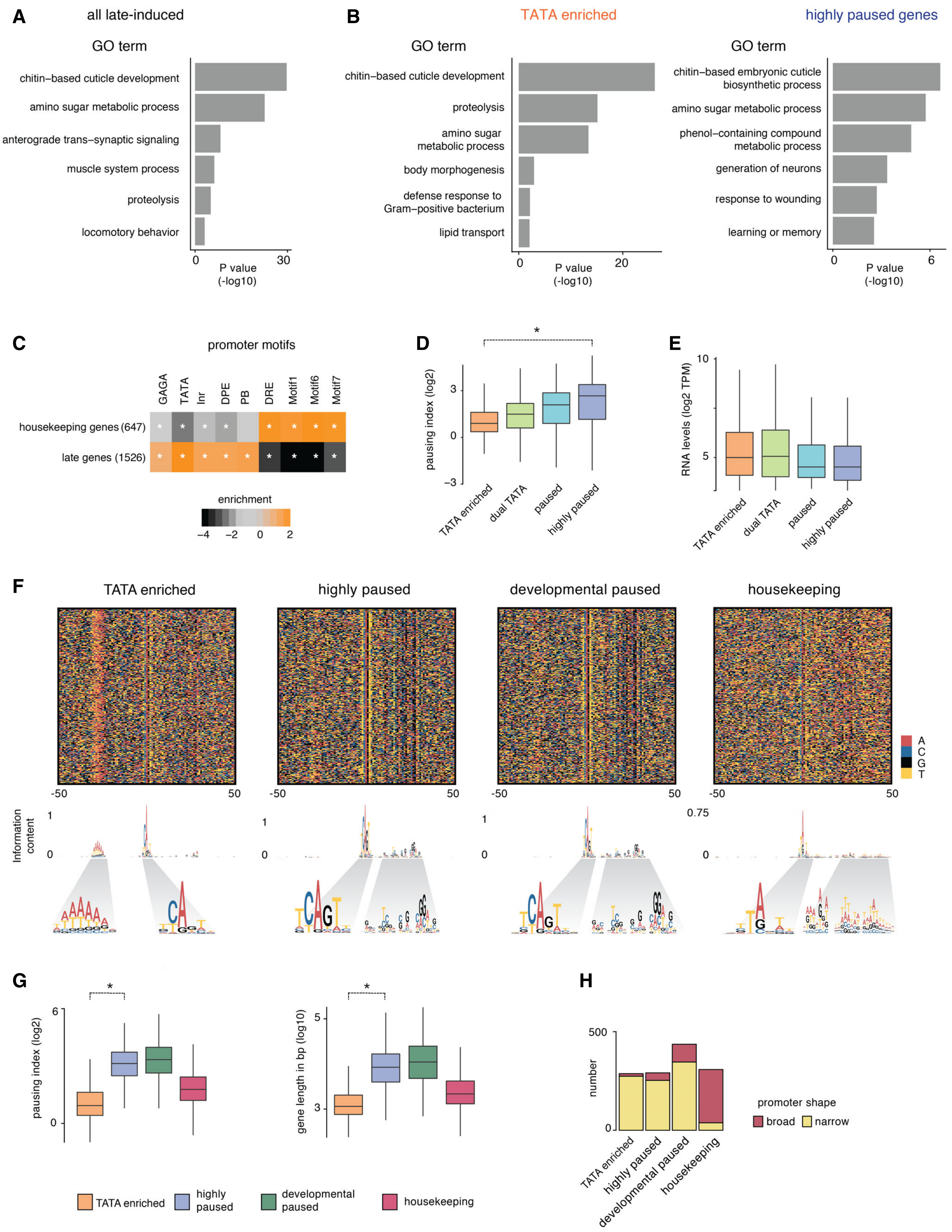

Figure EV1.

© 2021 The Authors

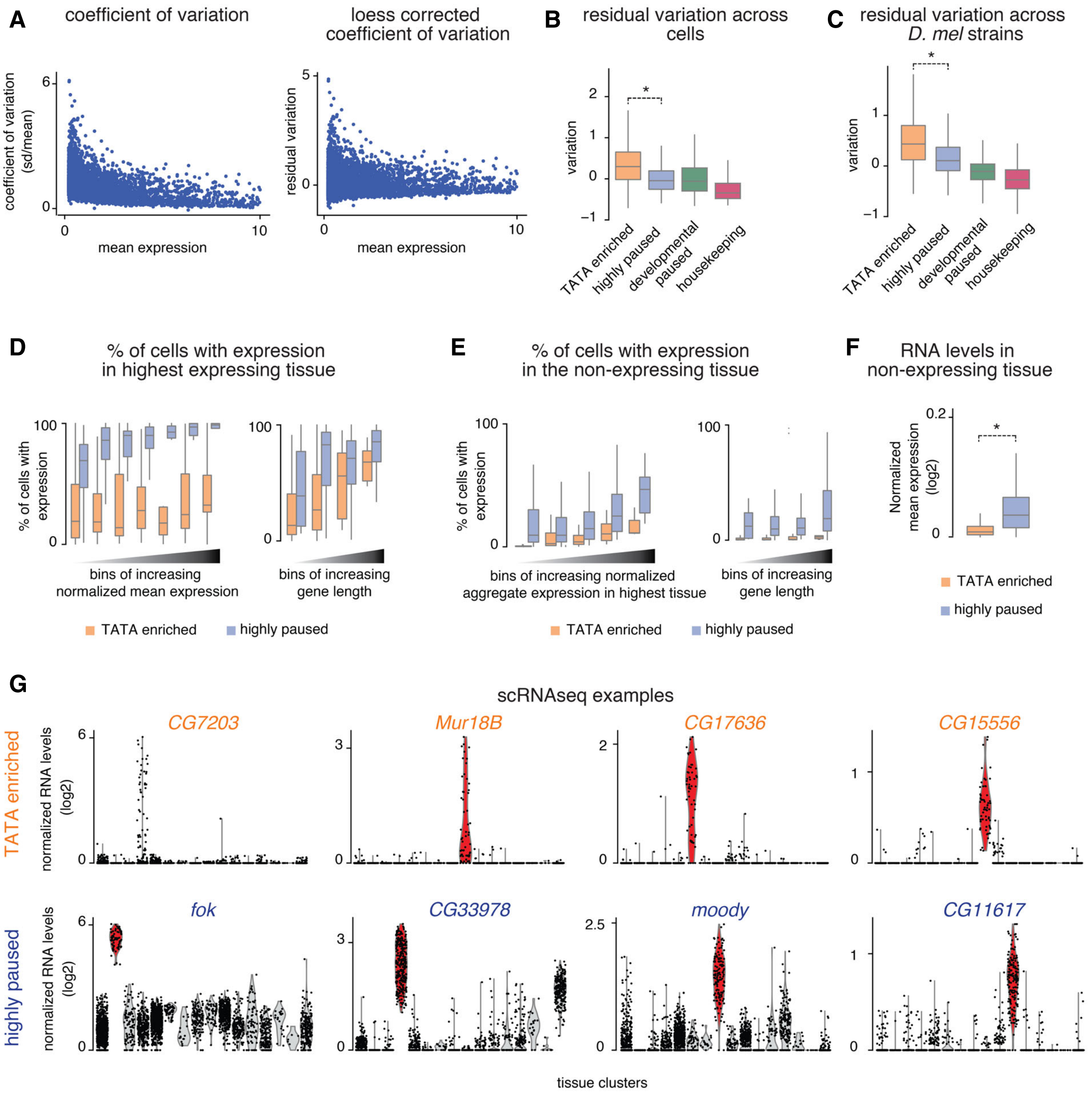
A
coefficient of variation
coefficient of variation (sd/mean)
mean expression
loess corrected coefficient of variation
residual variation
mean expression
B
residual variation across cells
variation
TATA enriched
highly paused
developmental paused
housekeeping
C
residual variation across D. mel strains
variation
TATA enriched
highly paused
developmental paused
housekeeping
D
% of cells with expression in highest expressing tissue
% of cells with expression
bins of increasing normalized mean expression
bins of increasing gene length
TATA enriched
highly paused
E
% of cells with expression in the non-expressing tissue
% of cells with expression
bins of increasing normalized aggregate expression in highest tissue
bins of increasing gene length
TATA enriched
highly paused
F
RNA levels in non-expressing tissue
Normalized mean expression (log2)
TATA enriched
highly paused
G
scRNAseq examples
TATA enriched
normalized RNA levels (log2)
CG7203
Mur18B
CG17636
CG15556
highly paused
normalized RNA levels (log2)
fok
CG33978
moody
CG11617
tissue clusters

Figure EV2.

Figure EV2. Differences in the scRNA-seq expression profiles of late-induced effector gene groups.

A Since the coefficient of variation of gene expression (standard deviation/mean) shows a dependency on mean expression (left), it was corrected by loess regression (right).

B The coefficient of variation after correcting for mean expression is lower for the paused genes compared with the TATA genes (Wilcoxon two-sided test, $*P < 10^{-15}$).

C The loess-corrected coefficient of variation of gene expression across different isogenic lines of *Drosophila melanogaster* from the DGRP collection (Sigalova *et al*, 2020) also shows that genes from the TATA-enriched group show higher variability compared to the paused genes (Wilcoxon two-sided test, $*P < 10^{-15}$).

D The frequency of cells with detectable expression (> one read) in the highest expressing tissue (different for each gene) was calculated for all genes in the different effector gene groups. The median frequency of expressing cells was higher for the highly paused genes compared with the TATA genes, stratified by the mean gene expression levels (left) and gene length (right). Across bins, the median frequency of cells with detectable expression was higher for the paused genes compared with the TATA genes.

E The frequency of cells with detectable expression (> one read) in the five least expressing tissues (different for each gene) was calculated for all genes in the different effector gene groups, stratified by total expression in the expressing tissues for each gene (left) and by gene length (right). The median frequency of cells with detectable expression was higher across bins for the highly paused genes compared with the TATA genes.

F Mean normalized RNA levels in the five least expressing tissues (different for each gene) were calculated for all genes in the different effector gene groups. The mean normalized RNA levels were higher for the highly paused genes compared with the TATA genes in the non-expressing tissues (Wilcoxon two-sided test, $*P < 10^{-15}$).

G Expression profiles of various genes from the TATA-enriched and highly paused genes groups show that the TATA-enriched group tends to have noisy expression without detectable background expression in non-expressing tissues, while the highly paused genes tend to show very robust expression in the expressing tissues with high background expression in the non-expressing tissues. Box plots in all panels show the median as the central line, the first and the third quartiles as the box, and the upper and lower whiskers extend from the quartile box to the largest/smallest value within 1.5 times of the interquartile range.

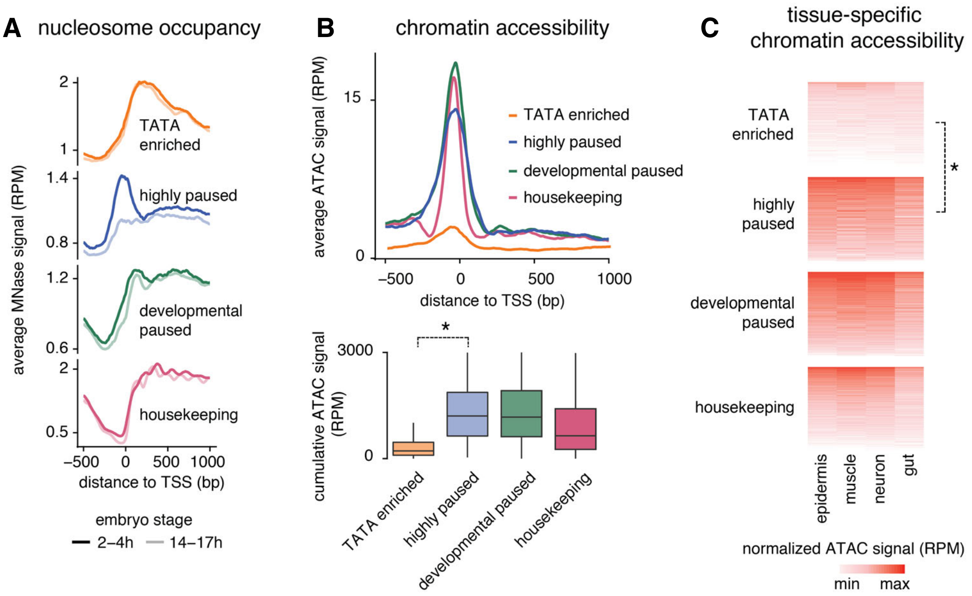

Figure EV3. Differences in accessibility at late-induced effector gene groups.

A Average read-count normalized MNase signal (RPM) from 2 to 4 h and 14 to 17 h embryos are shown at the different effector gene promoter groups and developmental and housekeeping genes.

B Chromatin accessibility is shown as the average read-count normalized ATAC-seq signal (RPM) from 14 to 17 h for each promoter group. TATA-enriched genes show lower accessibility at the promoter region than the highly paused effector genes or developmental genes. Box plots below quantifying the reads from 150 bp upstream of the TSS to the TSS confirm the lower accessibility of TATA genes compared with the paused genes (Wilcoxon two-sided test $*P < 10^{-15}$). Box plots show the median as the central line, the first and the third quartiles as the box, and the upper and lower whiskers extend from the quartile box to the largest/smallest value within 1.5 times of the interquartile range.

C Read-count normalized ATAC signals (RPM) from different tissues were calculated for each gene from 150 bp upstream of the TSS to the TSS. TATA-enriched genes show lower average accessibility across all tissues compared with the highly paused effector genes or developmental genes (Wilcoxon two-sided test, $*P < 10^{-15}$).

 © 2021 The Authors
